# Comparative Transcriptional Network Modeling of Three PPAR-α/γ Co-Agonists Reveals Distinct Metabolic Gene Signatures in Primary Human Hepatocytes

**DOI:** 10.1371/journal.pone.0035012

**Published:** 2012-04-13

**Authors:** Renée Deehan, Pia Maerz-Weiss, Natalie L. Catlett, Guido Steiner, Ben Wong, Matthew B. Wright, Gil Blander, Keith O. Elliston, William Ladd, Maria Bobadilla, Jacques Mizrahi, Carolina Haefliger, Alan Edgar

**Affiliations:** 1 Selventa, Cambridge, Massachusetts, United States of America; 2 F. Hoffmann-La Roche AG, Basel, Switzerland; National Institutes of Health, United States of America

## Abstract

**Aims:**

To compare the molecular and biologic signatures of a balanced dual peroxisome proliferator-activated receptor (PPAR)-α/γ agonist, aleglitazar, with tesaglitazar (a dual PPAR-α/γ agonist) or a combination of pioglitazone (Pio; PPAR-γ agonist) and fenofibrate (Feno; PPAR-α agonist) in human hepatocytes.

**Methods and Results:**

Gene expression microarray profiles were obtained from primary human hepatocytes treated with EC_50_-aligned low, medium and high concentrations of the three treatments. A systems biology approach, Causal Network Modeling, was used to model the data to infer upstream molecular mechanisms that may explain the observed changes in gene expression. Aleglitazar, tesaglitazar and Pio/Feno each induced unique transcriptional signatures, despite comparable core PPAR signaling. Although all treatments inferred qualitatively similar PPAR-α signaling, aleglitazar was inferred to have greater effects on high- and low-density lipoprotein cholesterol levels than tesaglitazar and Pio/Feno, due to a greater number of gene expression changes in pathways related to high-density and low-density lipoprotein metabolism. Distinct transcriptional and biologic signatures were also inferred for stress responses, which appeared to be less affected by aleglitazar than the comparators. In particular, Pio/Feno was inferred to increase NFE2L2 activity, a key component of the stress response pathway, while aleglitazar had no significant effect. All treatments were inferred to decrease proliferative signaling.

**Conclusions:**

Aleglitazar induces transcriptional signatures related to lipid parameters and stress responses that are unique from other dual PPAR-α/γ treatments. This may underlie observed favorable changes in lipid profiles in animal and clinical studies with aleglitazar and suggests a differentiated gene profile compared with other dual PPAR-α/γ agonist treatments.

## Introduction

The peroxisome proliferator-activated receptors (PPARs) are nuclear hormone receptors that function as transcription factors, regulating the expression of genes involved in the metabolism of fatty acids and carbohydrates. PPAR-α is highly expressed in the liver, kidney and skeletal muscle. Fibrates, which are weak PPAR-α agonists, alter hepatic energy metabolism that, in part, lowers plasma triglyceride (TG) levels and slightly increases high-density lipoprotein cholesterol (HDL-C) [1], [2]. PPAR-γ is highly expressed in adipocytes and, to a lesser extent, in other tissues, including the liver. PPAR-γ agonists, such as pioglitazone and rosiglitazone, improve insulin sensitivity, in part by reducing adipose TG lipolysis [1]. PPAR-α and PPAR-γ have also been shown to have anti-inflammatory properties in insulin-resistant states, leading to improvements in levels of cardiovascular (CV) risk markers [3]–[5].

Drugs that activate both PPAR-α and PPAR-γ may be expected to capture both the favorable changes in lipid profiles and the glycemic benefits, and have been pursued for their therapeutic potential to reduce CV risk associated with type 2 diabetes mellitus (T2DM). However, several previous dual PPAR-α/γ agonists were discontinued due to compound-specific side effects: tesaglitazar due to data suggesting irreversible impairment of renal function [6], [7] and muraglitazar due to concerns regarding increased CV risk [8]. The lessons emerging from these experiences are that each PPAR-targeted drug is different and that agonist profile alone may not reflect the underlying biology that defines the balance between efficacy and safety of specific molecules. In support of this notion, a recent report has shown that phosphorylation of PPAR-γ by cyclin-dependent kinase 5 is an important determinant of adipose insulin sensitivity [9]. Selective recruitment of co-activators or co-repressors may provide additional mechanisms whereby different PPAR-targeted drugs can differentially activate target pathways.

Aleglitazar is a novel PPAR-α/γ agonist that was designed to provide balanced activity on both receptors. Preclinical studies have shown that aleglitazar potently decreases blood glucose and insulin resistance and improves plasma lipids in animal models [10], [11]. In a Phase II clinical trial in patients with T2DM, aleglitazar provided effective glycemic control, improved lipid parameters, blood pressure and markers of CV risk, and was well tolerated [12]. Aleglitazar is currently under investigation in a large Phase III outcomes trial (ALECARDIO) evaluating its effect on CV mortality and morbidity in post-acute coronary syndrome patients with T2DM [13].

Since emerging evidence suggests that each PPAR drug has a unique efficacy and safety profile, we investigated the molecular effects of two different dual PPAR-α/γ agonists (aleglitazar and tesaglitazar) and a combination of a PPAR-γ and PPAR-α agonist (pioglitazone plus fenofibrate [Pio/Feno]) in human hepatocytes using whole-genome microarray studies in conjunction with a systems biology approach, Causal Network Modeling (CNM). Gene-expression profiling studies provide a broad, unbiased view of the transcriptional response to drug treatment, but the large amounts of data generated often require systems-based analytics for comprehensive interpretation. One common approach is to identify differentially expressed genes whose corresponding gene products act in biologic pathways of interest and extrapolate the biologic changes that may result from the changes in gene expression. Enrichment statistics may also be used to determine over-representation of genes involved in specific pathways in the set of differentially expressed genes [14]. This approach relies on the assumption that changes in gene expression directly correlate with changes in protein activity and is thereby limited by the validity of this assumption, as well as the inability to reason downstream of genes of unknown function.

CNM is a complementary approach that uses reverse-engineering principles to infer the activity states of signaling mechanisms that lead to the observed changes in gene expression. These inferences, called “hypotheses”, are then evaluated statistically and those that meet predefined statistical criteria are assessed for biological integrity. Examples of this vetting process include the following interrogations: determining whether an inferred change in the activity state of a biological mechanism or pathway is consistent with what would be expected in the context of the experiment under investigation, and examining whether the inferred mechanism is known to be expressed in the tissue under study. Causal links between different signaling mechanisms are also investigated and used to build models for disease states or therapeutic responsiveness, discover mechanisms of action, assess biomarkers for patient stratification or, as done here, differentiate the molecular effects induced by multiple treatments. This data-driven approach has been successfully applied to confirm or propose novel hypotheses, to identify molecular mechanisms involved in diverse biological processes *in vitro* [15]–[17] and *in vivo* [18], [19], and including discovery of potential biomarkers of clinical response to therapeutic intervention [19]. Example applications of CNM include models built that describe pulmonary proliferative and cellular stress processes [16], [17], hypoxia-induced hemangiosarcoma [20], the role of sirtuin-1 in human keratinocyte differentiation [16] and response to drug treatment [15], [18], [19].

The aim of the present CNM study was to compare the effects of aleglitazar with tesaglitazar and with Pio/Feno in a primary human hepatocyte model system to elucidate the molecular pathways modulated by aleglitazar and comparator treatments. Our study revealed that aleglitazar has caused a distinct gene signature profile compared with other dual PPAR-α/γ agonist treatments that likely underlies the observed favorable changes in lipid profiles in animal and clinical studies [10]–[12].

## Methods

### Cell culture

Primary human hepatocytes (Hepacult GmbH, Regensburg, Germany) were seeded at a density of 0.15×10^6^/cm^2^ in six-well plates. They were then incubated for 6 hours at 37°C in a 5% carbon dioxide-humidified atmosphere in 1.5 ml of William's Medium E + 2 mM L-glutamine (Sigma-Aldrich Corp., St Louis, MO, USA) containing aleglitazar (0.013, 0.064 or 0.32 µM), tesaglitazar (1.44, 7.2 or 36.0 µM), Pio/Feno (6.0/0.11, 30.0/0.56 or 150.0/2.8 µM) or vehicle (0.1% dimethyl sulfoxide). The concentrations were chosen based on *in vitro* transactivation data providing EC_50_-aligned low, medium and high concentrations of each of the three treatments, such that there was similar receptor activation across treatments at each concentration level.

### RNA preparation

Total RNA was extracted with TRI Reagent® (Molecular Research Center, Inc., Cincinnati, OH, USA) according to the manufacturer's instructions and RNA samples were stored at −80°C. Total RNA was quantified on a NanoDrop™ spectrophotometer (NanoDrop Technologies Inc., Wilmington, DE, USA) and RNA integrity was determined on an Agilent 2100 Bioanalyzer (Agilent Technologies, Palo Alto, CA, USA).

### Gene-expression microarray analysis

Total RNA (1 µg/sample) was converted to complementary DNA (cDNA) using the low-RNA input fluorescent linear amplification kit (Agilent Technologies). cDNA was converted to cRNA and biotinylated using biotin-LC vinyl CTP. Amplified and biotinylated cRNA was quantified and quality checked by formaldehyde agarose gel electrophoresis. After statistical randomization of sample order, labeled cRNA (1.5 µg/sample) was used for array hybridization using the Whole-Genome Gene Expression Direct Hybridization Assay and Illumina Human-6 Expression BeadChips version 3 arrays (Illumina Inc., San Diego, CA, USA). Staining and washing steps were performed as suggested by the manufacturer. BeadChips were scanned using an Illumina BeadArray® reader (Illumina Inc.) and raw data were extracted using BeadStudio© software (version 3.1.3.0) with Gene Expression Module 3.4.0 (Illumina Inc.).

Gene-expression profiles were obtained using EC_50_-aligned low, medium and high concentrations of the three treatments as specified above, such that there was similar receptor activation across treatments at each concentration level. Transcript data were analyzed using five replicates of each treatment concentration in order to identify significant changes in gene expression between the treatment and the vehicle control (RNA state changes).

### Determination of significant RNA expression changes

RNA expression data were analyzed using the “affy” and “limma” packages of the Bioconductor suite of microarray analysis tools available for the R statistical package [21], [22]. Robust microarray analysis background correction and quantile normalization were used to generate microarray expression values. Prior to analysis of changed genes, data were visualized using principal-components analysis and hierarchical clustering to identify outlier samples. An overall linear model was fitted to the data for all sample groups and specific contrasts of interest were evaluated to generate raw *p*-values for each probe set on the expression array [23]. The Benjamini–Hochberg false discovery rate method [24] was then used to correct for multiple testing effects. Probe sets were considered to have changed qualitatively in a specific comparison if an adjusted *p*-value of 0.05 was obtained, their average expression intensity was >150 in any treatment group and they had an absolute change >1.3-fold. Genes represented by multiple probe sets were considered to have changed if at least one probe set was observed to change. Gene-expression changes that met these criteria were termed “statistically significant RNA state changes” and had the directional qualities of “up” or “down” (i.e. they can be upregulated or downregulated in response to treatment). We confirm that the microarray data is MIAME compliant and has been deposited in the GEO database (#GSE33152) http://www.ncbi.nlm.nih.gov/geo/query/acc.cgi?acc=GSE33152.

### Evaluation of signaling networks using Causal Network Modeling

The CNM platform uses a reverse engineering approach to automatically generate hypotheses that are statistically significant explanations of RNA state changes. The substrate for this approach is a knowledgebase comprised of qualitative causal relationships in a computable format, e.g. the transcriptional activity of PPAR-γ increases the RNA expression of insulin-induced gene 1 (*INSIG-1*) [25]. These cause and effect relationships are derived from the knowledgebase at the time of completion/analysis of the present study. That version contained biological interactions derived from 50,827 unique PubMed entries encompassing studies in human, mouse and rat. The human-specific assembly of this knowledgebase includes relationships defined both in human and in rodent studies with gene and proteins mapped to the human homologue. The current knowledgebase contains more than 100,000 concepts and molecular entities, and more than 400,000 causal relationships. Each hypothesis is ranked according to two probabilistic scoring metrics, richness and concordance, which examine distinct aspects of the probability of a hypothetical cause explaining a given number of RNA state changes. *Richness* is based on hypergeometric distribution and represents the probability that the number of observed RNA state changes connected to a given hypothesis could have occurred by chance alone. *Concordance* is based on binomial distribution and represents the probability that the number of observed RNA state changes that match the directionality of the hypothesis (e.g. increased or decreased kinase activity for a kinase and increased or decreased transcriptional activity for a transcription factor) could have occurred by chance alone. Stronger concordance indicates high confidence in the inferred directionality of the hypothesis. Those hypotheses with concordance *p*<0.1 as well as richness *p*<0.1 were considered to be statistically significant. Application of these significance values to randomly generated data produces less than 5% of the number of hypotheses meeting both significance criteria than are observed for experimental data with similar numbers of RNA state changes. Hypotheses were further investigated and prioritized by evaluation of their biologic relevance to the experimental context, whether they could be causally linked to phenotypes observed and processes relevant to PPAR-α/γ biology or metabolism, as evidenced in the literature, and if they were causally downstream of the experimental treatments. Hypotheses that were determined to be biologically relevant for the model were assessed for their relationship to other hypotheses of interest and then ordered causally into small models based on causal relationships in the knowledgebase or additional relevant knowledge identified in the literature. Several examples of applications of this technology and associated methodology have been published [15]–[19], [26].

### Explanation of hypothesis tables

**Figure 1:**
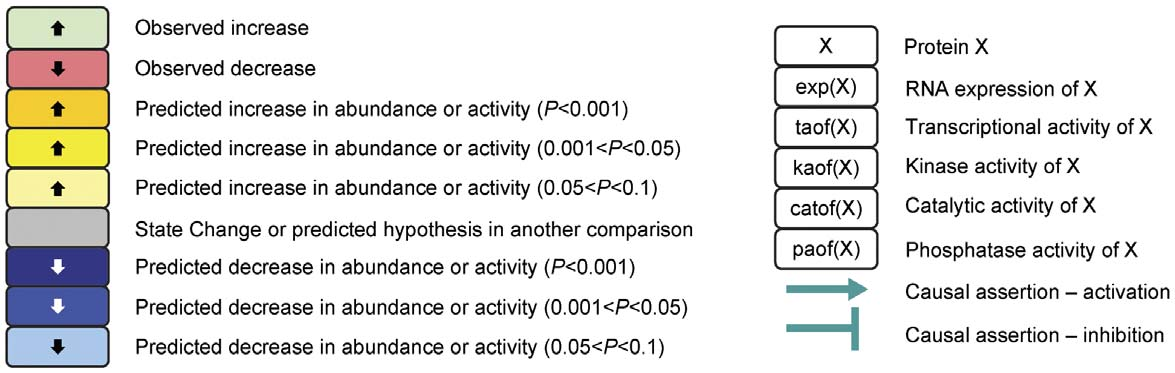
Key for figures and hypothesis tables. Green and red boxes = observed increases and decreases, respectively, in RNA expression of a given gene. Gene expression is depicted as “exp” followed by the National Center for Biotechnology Information gene symbol in parentheses. Yellow and blue boxes = biologic processes or protein activities that are inferred by the CNM approach to increase or decrease upon treatment, respectively. Darker shades of yellow or blue = stronger concordance. Numbers in the blue or yellow boxes indicate the number of gene expression state changes that support the inference: negative numbers indicate an inferred decrease and positive numbers indicate an inferred increase. Numbers in the green or red boxes indicate the log fold change.

As illustrated in *Figure 1*, green and red boxes indicate observed increases or decreases in RNA expression of a given gene, respectively. Gene expression is depicted as “exp” followed by the National Center for Biotechnology Information gene symbol in parentheses. Yellow and blue boxes indicate biologic processes or protein activities that are inferred by reverse causal reasoning to increase or decrease upon treatment, respectively. Darker shades of yellow or blue indicate stronger concordance. Numbers in the boxes indicate the number of gene-expression state changes that support the inference. Negative numbers indicate an inferred decrease, while positive numbers indicate an inferred increase.

## Results

### State change analysis

**Figure 2:**
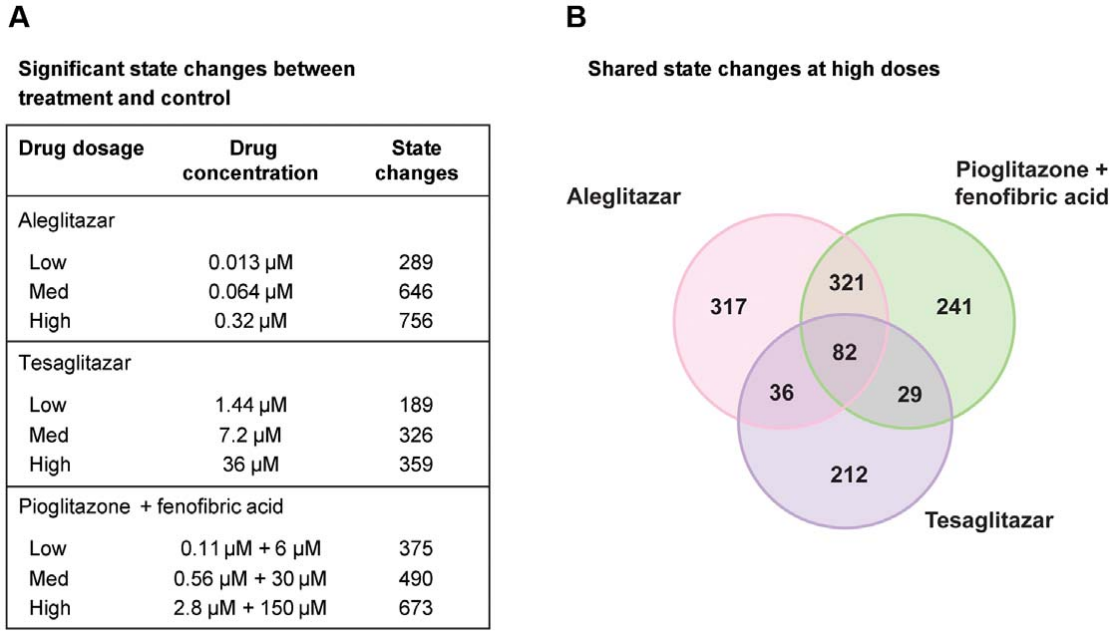
Treatment-induced RNA and gene expression changes. (**A**) Total number of RNA state changes (up- or downregulation) in human hepatocytes treated with aleglitazar, tesaglitazar or Pio/Feno. (**B**) Unique and shared gene expression following treatment with aleglitazar, tesaglitazar or Pio/Feno.

**Figure 3:**
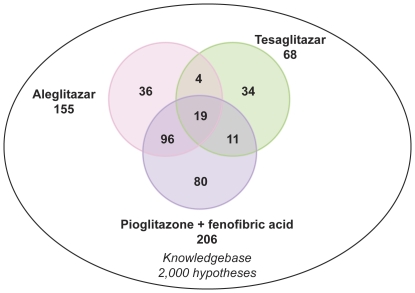
Molecular pathways modulated in response to treatment with aleglitazar, tesaglitazar or Pio/Feno. Number of RNA expression changes observed following treatment with aleglitazar, tesaglitazar or Pio/Feno. Of the 2,000 unique mechanisms represented in the knowledgebase, 280 were considered statistically significant in at least one treatment condition and are designated as “hypotheses”.

**Figure 4:**
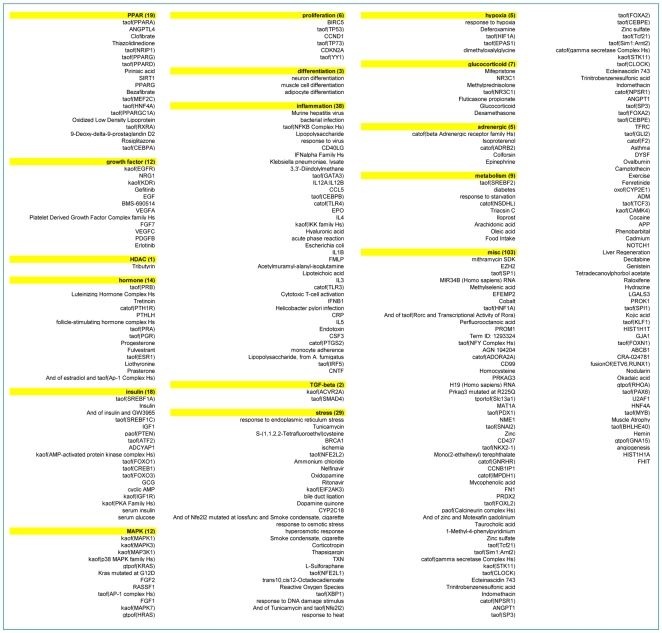
Clustering of the hypotheses into broader biologic processes/pathways. Numbers (in the boxes) indicate the number of genes/hypotheses in molecular pathway clusters.

Aleglitazar, tesaglitazar and Pio/Feno treatment of primary human hepatocytes resulted in both shared and distinct RNA expression state changes (*Figure 2*). At the high drug dosage, only 82 out of 1,238 (6.6%) total state changes observed were induced by all three agonist treatments (*Figure 2B*). There were only 29 (2.3%) and 36 (2.9%) state changes that were shared by tesaglitazar and Pio/Feno or aleglitazar and tesaglitazar treatments, respectively (*Figure 2B*). CNM was then used to generate statistically significant inferences (i.e. those with both richness and concordance *p*-values<0.1) of the molecular signaling networks underlying these RNA expression changes, and to provide explanations for the RNA expression changes in each treatment group. *Figure 3* shows the inferred biologic pathways affected by each treatment. The 2,000 hypotheses/molecular predictions represented in the knowledgebase were scored across all treatment groups; 280 unique predictions were identified and 276 of these met the criteria for statistical significance in at least one of the three high drug dosage treatments (*Figure 3*) and since a consistent direction of effect was inferred these were considered to be hypotheses. The molecular pathways represented by these hypotheses were then clustered under broader biologic processes, e.g. PPAR signaling, metabolic pathways, inflammation and stress, insulin signaling, cell proliferation and mitogen-activated protein kinase pathways (*Figure 4*). The following sections detail the biologic signatures that were inferred to be most robustly modified by the three treatments.

### PPAR-α signaling

**Figure 5:**
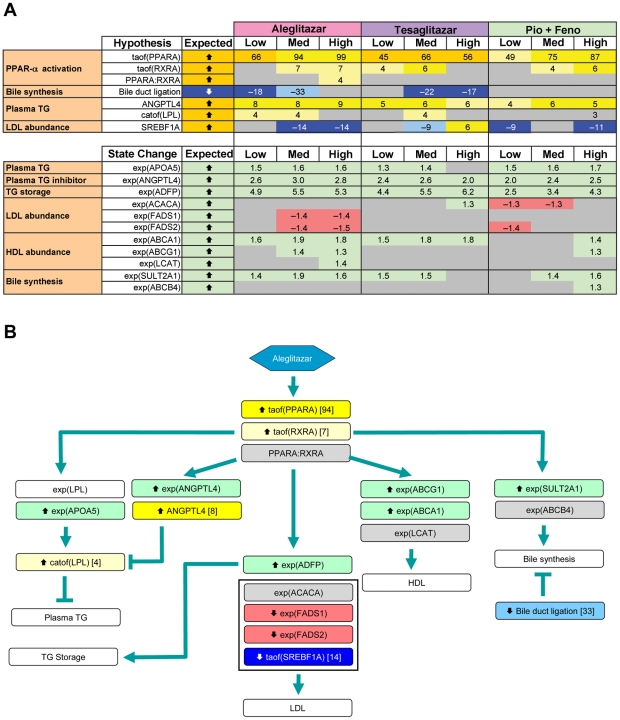
PPAR-α signaling, lipid metabolism-associated RNA changes and inferred signaling pathways. (**A**) Observed RNA state changes and Causal Network Modeling–based predictions of PPAR-α signaling and lipid metabolism upon treatment with aleglitazar, tesaglitazar or Pio/Feno. See *Figure 2A* for treatment dosages. “Bile duct ligation” is known as a “proxy” hypothesis and describes the finding that changes in gene expression observed in the current experiments are consistent with gene expression modulations in other experiments where bile duct ligation was the perturbation. Similarly “Response to osmotic stress”, “Reponse to shear stress”, “Corticotropin”, “Response to DNA damage stimulus”, “shear stress” and “Response to stress-inducing agents” (e.g. nelfinavir) are also proxy hypotheses. (**B**) Molecular signaling pathways, predicted by RNA state changes shown in panel A, that could lead to changes in lipid parameters. Depiction is based on data derived from treatment with the medium concentration of aleglitazar. Numbers in the blue or yellow boxes indicate the number of gene expression state changes that support the inference: negative numbers indicate an inferred decrease and positive numbers indicate an inferred increase. Numbers in the green or red boxes indicate the log fold change. HDL, high-density lipoprotein; LDL, low-density lipoprotein; Med, medium; TG, triglycerides.

In the liver, PPAR-α activation modulates lipid metabolism and lipoprotein synthesis and secretion, affecting TGs, HDL-C synthesis and regulation of bile acid synthesis and detoxification [27]. CNM identified qualitatively similar PPAR-α signaling when comparing the medium concentrations of all three treatments (*Figure 5A*), with increased PPAR-α transcriptional activity supported by broadly similar gene expression changes (94, 66 and 75 for aleglitazar, tesaglitazar and Pio/Feno, respectively) (*Figure 5A*).

### Lipid metabolic pathways

All three treatments may promote activation of signaling pathways related to a decrease in plasma TG levels (*Figure 5*). Aleglitazar, tesaglitazar and Pio/Feno each upregulated RNA expression of adipose differentiation-related protein (ADFP), angiopoietin-like 4 (ANGPTL4) and apolipoprotein A-V (Apo-AV) to similar degrees. In addition, CNM infers that the abundance of ANGPTL4 itself is increased. Transcriptional control of all three of these genes is coordinately modulated by PPAR-α [28]–[30]. ADFP is a lipid droplet coat protein present in non-adipose cells that do not express perilipin [28]. It is broadly distributed in non-adipogenic liver and muscle tissues and plays a surfactant role at the lipid droplet surface, mediating triacyl-glycerol packaging [31]. ANGPTL4 and Apo-AV affect plasma TG metabolism in part by either inhibiting or potentiating the activity of lipoprotein lipase, which mediates TG hydrolysis [32]–[35].

In contrast to the similar treatment effects observed in pathways related to TG metabolism, CNM inferred that aleglitazar increases expression of signaling pathways related to HDL-C metabolism to a greater extent than tesaglitazar or Pio/Feno. In general, aleglitazar treatment induced more changes in gene expression related to HDL-C metabolism than tesaglitazar and Pio/Feno, particularly at the high concentration (*Figure 5A*). ATP-binding cassette transporter proteins A1 (ABCA1) and G1 (ABCG1) promote HDL formation by increasing cholesterol efflux to pre-β and α HDL particles [36]. Aleglitazar significantly increased *ABCA1* expression at all concentrations and *ABCG1* expression at the medium and high concentrations (*Figure 5A*). In contrast, tesaglitazar increased *ABCA1* expression at all concentrations but did not have a significant effect on *ABCG1*, while Pio/Feno increased *ABCA1* and *ABCG1* expression only at the highest concentrations. The greater effect of aleglitazar on *ABCA1* and *ABCG1* versus tesaglitazar and Pio/Feno suggests that aleglitazar may increase HDL-C to a greater extent, with a superior effect on reverse cholesterol transport than tesaglitazar or Pio/Feno.

The three treatments used in this study were also inferred to have differential effects on pathways related to low-density lipoprotein (LDL) synthesis, as exemplified by their effects on sterol regulatory element binding transcription factor 1A (SREBF1A) activity and fatty acid desaturase 1 and 2 (*FADS1/2*) mRNA expression [37]. Aleglitazar and Pio/Feno were inferred to significantly decrease SREBF1A activity (at the medium and high concentrations of aleglitazar and the low and high concentrations of Pio/Feno), while tesaglitazar decreased SREBF1A activity at the medium concentration and increased it at the high concentration (*Figure 5A*). Aleglitazar also significantly decreased the expression of both *FADS1* and *FADS2* at medium and high concentrations, while Pio/Feno decreased *FADS2* expression only at the low concentration. Tesaglitazar had no effect. These data suggest that aleglitazar treatment may more strongly decrease LDL than tesaglitazar.

Based on the above inferences, it was possible to develop a model that represents how aleglitazar may affect a number of molecular signaling pathways involved in lipid metabolism (*Figure 5B*).

### Cellular stress response

**Figure 6:**
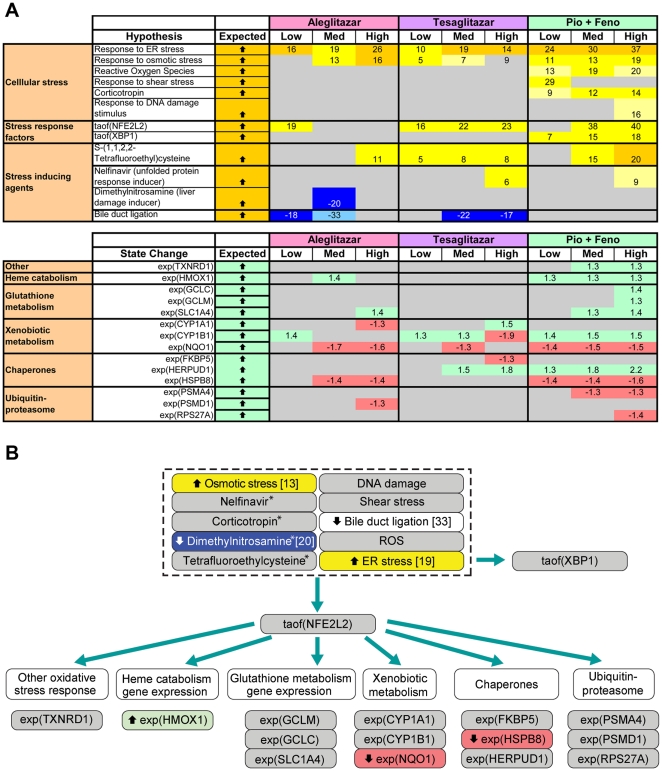
Cellular stress response-associated RNA changes and inferred signaling pathways. (**A**) Biologic processes associated with the cellular stress response inferred to increase or decrease upon treatment with aleglitazar, tesaglitazar or Pio/Feno. See *Figure 2A* for treatment dosages. As in *Figure 5A*, proxy hypotheses are shown. (**B**) Molecular signaling pathway, predicted by RNA state changes shown in panel A, that could lead to changes in the stress response. Numbers in the blue or yellow boxes indicate the number of gene expression state changes that support the inference: negative numbers indicate an inferred decrease and positive numbers indicate an inferred increase. Numbers in the green or red boxes indicate the log fold change. * Denotes genes/pathways responsive to the agent cited. ER, endoplasmic reticulum; Med, medium; ROS, reactive oxygen species.

CNM inferred a relatively weak cellular stress response following aleglitazar treatment compared with tesaglitazar and Pio/Feno (*Figure 6*). The activity of nuclear factor (erythroid-derived 2)-like 2 (NFE2L2), a transcription factor activated by, and involved in, mediating oxidative and xenobiotic stresses [38], was inferred to increase for the medium and high concentrations of tesaglitazar and Pio/Feno (*Figure 6A*). These increases are supported by 22 and 23 (tesaglitazar) and 38 and 40 (Pio/Feno) gene expression changes, respectively. In contrast, statistically significant changes in NFE2L2 activity were inferred only for the low concentration of aleglitazar, while medium or high concentrations of aleglitazar had no effect. This weaker stress response to aleglitazar was further supported by the absence of an inferred effect of aleglitazar on nelfinavir-responsive genes (a proxy for unfolded protein response and cellular stress) [39] at any concentration, which was evident with the high concentrations of both tesaglitazar and Pio/Feno. In addition, only aleglitazar was inferred to decrease dimethylnitrosamine-responsive genes (a proxy for liver damage) [40], consistent with the notion that aleglitazar induces a minimal stress response. These inferences allowed the modeling of molecular signaling pathways that could lead to stress response, and of the potential effects of aleglitazar treatment on these molecular mechanisms (*Figure 6B*).

### Insulin signaling and cellular proliferation pathways

**Figure 7:**
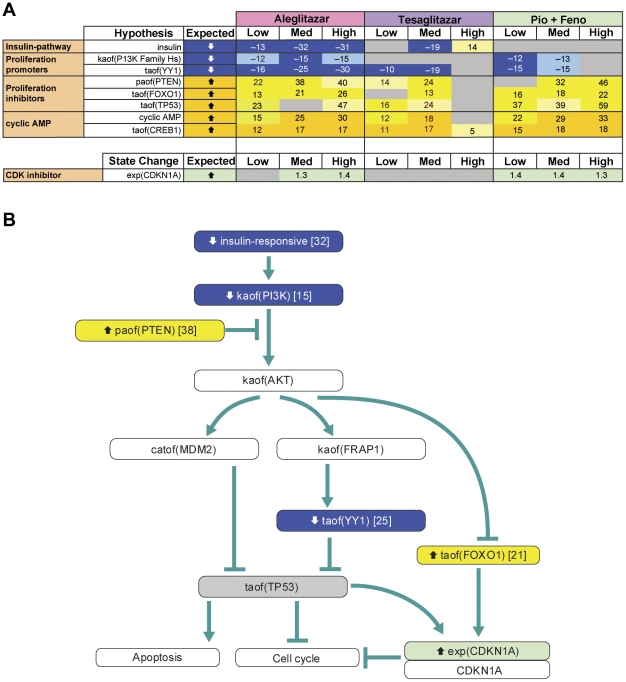
Insulin signaling and biologic response-associated RNA changes and inferred signaling pathways. (**A**) Observed RNA state changes related to insulin signaling and biologic processes inferred to increase or decrease upon treatment with aleglitazar, tesaglitazar or Pio/Feno. See *Figure 2A* for treatment dosages. (**B**) Molecular signaling pathway that could lead to changes in insulin signaling to decrease cellular proliferation. Depiction is based on data derived from treatment with the medium concentration of aleglitazar. Numbers in the blue or yellow boxes indicate the number of gene expression state changes that support the inference: negative numbers indicate an inferred decrease and positive numbers indicate an inferred increase. Numbers in the green or red boxes indicate the log fold change. AMP, adenosine monophosphate; CDK, cyclin-dependent kinase; Med, medium.

CNM inferred that aleglitazar would decrease expression of insulin-responsive genes at all three concentrations (*Figure 7A*), while tesaglitazar would have differing effects at the medium and high concentrations (decreasing and increasing insulin-responsive genes/pathways, respectively) and Pio/Feno would have no significant effect at any concentration. In addition, CNM inferred that all three treatments would affect multiple pathways involved in decreased cell proliferation and survival signaling. All three concentrations of aleglitazar were inferred to decrease the activity of phosphoinositide-3-kinase (PI3K) and Yin-Yang 1 (YY1) proteins, which leads to proliferation and survival [41], [42], while tesaglitazar had no significant inferred effect on PI3K at any concentration and Pio/Feno decreased PI3K at the low and medium concentrations. Similarly, only the low and medium concentrations of tesaglitazar and Pio/Feno had inferred effects on YY1 activity. All three treatments were also inferred to increase the activities of signaling that lead to inhibition of proliferation, namely phosphatase activity of phosphatase and tensin homolog (*PTEN*) and transcriptional activity of forkhead box protein (FOXO1) and tumor protein 53 (p53) [43]. Consistent with the inferred changes in insulin signaling, there was also an inferred increase in cyclic AMP and cAMP**-**responsive element-binding protein 1 with all treatments, although the high concentration of tesaglitazar failed to have any effect. Additionally, both aleglitazar and Pio/Feno increased cyclin-dependent kinase inhibitor 1A (*CDKN1A*) expression, which leads to cell cycle arrest [44], while tesaglitazar had no significant effect at any concentration. The molecular signaling pathway that could result in inhibition of proliferation by these treatments is shown in *Figure 7B* and is colored for measurements and inferences from the medium concentration of aleglitazar.

## Discussion

Analysis of gene transcript data from human hepatocytes using CNM revealed that the balanced dual PPAR-α/γ agonist aleglitazar exerts molecular effects that are distinct from the effects of tesaglitazar (a dual PPAR-α/γ agonist) or combination Pio/Feno (a PPAR-γ agonist/PPAR-α agonist treatment), particularly with regard to lipid metabolism and the cellular stress response.

All three treatments led to qualitatively similar PPAR-α signaling, and to increases in genes related to decreases in TG levels. The observed increase in *ADFP* mRNA expression is consistent with published literature, which has demonstrated that PPAR agonists can upregulate *ADFP* mRNA *in vitro* and *in vivo* [28], [45]. Although the exact function of *ADFP* is unclear, it is hypothesized that it may play a crucial role in sequestering TGs in cytosolic lipid droplets for storage [28]. The observed increases in *ANGPTL4* and *Apo-AV* mRNA are consistent with previous studies, which have shown that PPAR-α agonists increase *ANGPTL4* and *Apo-AV* expression [1], [2], [45]. ANGPTL4 and Apo-AV collectively regulate metabolism by inhibiting and potentiating the activity of lipoprotein lipase (which mediates TG hydrolysis) [32]–[35], and it is the composite interplay between these regulators that contributes to the decrease in plasma TGs. Furthermore, mutations in *ANGPTL4* have been shown to be significantly related to TG levels and, in some cases, to altered CV risk [46]. The observed changes in the expression of genes related to decreased TGs are also consistent with clinical results showing that all treatments effectively decrease TG levels in patients with T2DM [6], [7], [12], [47], [48].

CNM inferred differences between the three treatments with regard to effects on HDL-C biosynthetic and metabolic pathways and with regard to expression of genes related to HDL synthesis. Aleglitazar was associated with greater increases in expression of genes related to HDL synthesis (*ABCA1* and *ABCG1*) than either tesaglitazar or Pio/Feno, which suggests that aleglitazar may increase HDL-C levels to a greater extent than tesaglitazar or Pio/Feno. This is consistent with the between-study comparison of clinical trial results in patients with T2DM for aleglitazar and tesaglitazar, which shows both treatments increase HDL-C, but tentatively suggests aleglitazar has a greater HDL-C effect (∼25% increase [12]) than tesaglitazar (∼17.9% increase [6]) from baseline. In addition, fibrates and glitazones have both shown more modest HDL-C effects in clinical trials [49], [50]. In the Phase II SYNCHRONY trial, aleglitazar exerted a greater effect on HDL-C compared with open-label Pio (∼25% vs ∼16%, respectively) [12]. Elucidation of the specific pathways that may underlie the differences between aleglitazar, tesaglitazar and Pio/Feno requires further investigation.

CNM also showed that aleglitazar is associated with greater inferred decreases in SREBF1A activity compared with tesaglitazar, supported by decreased mRNA expression of genes related to LDL synthesis (*FADS1/2*). These results suggest that aleglitazar may more effectively decrease LDL than tesaglitazar. Indeed, a previous study demonstrated mild to moderate induction of FADS1 with the PPAR agonists tesaglitazar and muraglitazar, respectively [45]. The greater relative magnitude of the changes in LDL inferred for aleglitazar in this study are also consistent with results from clinical studies, which have found that aleglitazar significantly decreases LDL levels in patients with T2DM [12]. Tesaglitazar was reported to decrease LDL in several studies [6], [47], [48], with one exception, in which tesaglitazar, in combination with insulin, increased LDL [7]. The greater magnitude of effect of aleglitazar versus Pio/Feno on LDL is also consistent with results from clinical studies, which have shown that both Pio and fibrates affect LDL particle size and density but have minimal effects on LDL levels [51], [52].

When cells are exposed to oxidative stress, electrophiles or other exogenous agents, a complex cascade of stress-inducible enzymes and related transcription factors is initiated. The cellular stress response induced by therapeutic intervention may have implications for drug safety. Deregulation of genes involved in the oxidative stress response (*HMOX1*, *POR*) has previously been shown in response to both PPAR-γ and dual PPAR-α/γ agonists [45]. In the present study, inferred changes in pathways contributing to the cellular stress response (*NFE2L2*, nelfinavir-responsive genes and dimethylnitrosamine-responsive genes) were less strongly induced by aleglitazar than by tesaglitazar or Pio/Feno. *NFE2L2* may lead to the induction of other genes that may, in turn, lead to changes in the stress response, such as heme catabolism gene expression, glutathione metabolism gene expression, xenobiotic metabolism, chaperones and the ubiquitin-proteasome pathway. Since tesaglitazar and Pio/Feno were inferred to more strongly induce *NFE2L2* than aleglitazar, aleglitazar may have less potential for toxic effects than tesaglitazar or Pio/Feno.

All three treatments exerted similar effects on mechanisms related to proliferative signaling, decreasing activity related to proliferation and survival (PI3K and YY1) and increasing activity related to apoptosis and decreased proliferation (PTEN, FOXO1 and p53). FOXO1 and p53 activity inhibit cell cycle progression and proliferation, in part through the increased expression of the cyclin-dependent kinase inhibitor p21 (*CIP1*/*WAF1*/*CDKN1A*). In addition to these components, changes in E2F family, RB1 and CDKN1A activity are usually inferred in association with experimentally observed inhibition or activation of the cell cycle [53]. Such supporting mechanisms were not inferred for any treatment in this study. Inferred changes in MYC or MYCN activity, which, in our experience, are also generally strongly associated with changes in proliferation, were also not observed in this study. Thus, interpretation of the effect of treatment on cell cycle progression must be made with caution in the absence of these supporting observations or direct studies. Similarly, inference of changes in glucose handling as a consequence of purported reductions in PI3K and increased PTEN activities must be made with caution.

Several limitations in interpretation of the results of this study should be acknowledged. The inferences of change in activity of genes/pathways, while drawn from a curated knowledgebase of previously published wet-laboratory experiments, are *in silico* predictions and must be independently validated in further investigations. A recent study by Rogue *et al.* revealed interindividual variability in the response of different human primary hepatocyte populations to different PPAR agonists [45]. However, that investigation employed very high concentrations of PPAR ligands (e.g. for tesaglitazar, 300 µM, >400-fold human therapeutic exposure [54]), much higher than the concentrations employed in our study (1.4 to 36 µM). Furthermore, Rogue *et al.* did not correct for multiple testing. The high concentrations of tesaglitazar in the study by Rogue *et al.* induced between 2,111 and 3,277 differentially expressed genes, representing >10% of all genes represented on the arrays. In contrast, we found only 189, 326 and 359 gene state changes at the low (1.4 µM), medium (7.2 µM) and high (36 µM) concentrations of tesaglitazar, respectively. We believe that use of pharmacologically relevant concentrations of ligands, a uniform cell population, short incubation times, sufficient biological replicates and rigorous data analysis (e.g. correction for multiple testing) is critical to ensure detection of specific gene signatures that allow rigorous comparative evaluation of differential effects of the different ligands. However, it is worth noting that despite the differences between the two studies Rogue *et al*. also concluded that individual PPAR agonists exhibit unique yet overlapping gene-expression profiles [45].

Based on the data presented here, distinct dual PPAR-α/γ agonist treatments induce unique transcriptional signatures, despite comparable core PPAR signaling. Using CNM, aleglitazar, a balanced dual PPAR-α/γ agonist, was shown to have a distinct gene activation profile compared with the dual PPAR-α/γ agonist tesaglitazar and concomitant Pio/Feno treatment. These observations may translate into benefits in dyslipidemia, as well as a differentiated safety profile for aleglitazar compared with other dual PPAR-α/γ agonists. Aleglitazar is currently under investigation in a large outcomes-driven Phase III study (ALECARDIO), evaluating its ability to reduce CV morbidity and mortality in post-acute coronary syndrome patients with T2DM [13].

## References

[1] Staels B, Fruchart JC (2005). Therapeutic roles of peroxisome proliferator-activated receptor agonists.. Diabetes.

[2] Tenenbaum A, Motro M, Fisman EZ (2005). Dual and pan-peroxisome proliferator-activated receptors (PPAR) co-agonism: the bezafibrate lessons.. Cardiovasc Diabetol.

[3] Pfutzner A, Marx N, Lubben G, Langenfeld M, Walcher D (2005). Improvement of cardiovascular risk markers by pioglitazone is independent from glycemic control: results from the pioneer study.. J Am Coll Cardiol.

[4] Delerive P, Fruchart JC, Staels B (2001). Peroxisome proliferator-activated receptors in inflammation control.. J Endocrinol.

[5] Barbier O, Torra IP, Duguay Y, Blanquart C, Fruchart JC (2002). Pleiotropic actions of peroxisome proliferator-activated receptors in lipid metabolism and atherosclerosis.. Arterioscler Thromb Vasc Biol.

[6] Bays H, McElhattan J, Bryzinski BS (2007). A double-blind, randomised trial of tesaglitazar versus pioglitazone in patients with type 2 diabetes mellitus.. Diab Vasc Dis Res.

[7] Ratner RE, Parikh S, Tou C (2007). Efficacy, safety and tolerability of tesaglitazar when added to the therapeutic regimen of poorly controlled insulin-treated patients with type 2 diabetes.. Diab Vasc Dis Res.

[8] Nissen SE, Wolski K, Topol EJ (2005). Effect of muraglitazar on death and major adverse cardiovascular events in patients with type 2 diabetes mellitus.. Jama.

[9] Choi JH, Banks AS, Estall JL, Kajimura S, Bostrom P (2010). Anti-diabetic drugs inhibit obesity-linked phosphorylation of PPARgamma by Cdk5.. Nature.

[10] Benardeau A, Benz J, Binggeli A, Blum D, Boehringer M (2009). Aleglitazar, a new, potent, and balanced dual PPARalpha/gamma agonist for the treatment of type II diabetes.. Bioorg Med Chem Lett.

[11] Hansen BC, Tigno XT, Benardeau A, Meyer M, Sebokova E (2011). Effects of aleglitazar, a balanced dual peroxisome proliferator-activated receptor alpha/gamma agonist on glycemic and lipid parameters in a primate model of the metabolic syndrome.. Cardiovasc Diabetol.

[12] Henry RR, Lincoff AM, Mudaliar S, Rabbia M, Chognot C (2009). Effect of the dual peroxisome proliferator-activated receptor-alpha/gamma agonist aleglitazar on risk of cardiovascular disease in patients with type 2 diabetes (SYNCHRONY): a phase II, randomised, dose-ranging study.. Lancet.

[13] ALECARDIO study. A study with aleglitazar in patients with a recent acute coronary syndrome and type 2 diabetes mellitus.. http://clinicaltrials.gov/ct2/show/NCT01042769.

[14] Huang da W, Sherman BT, Lempicki RA (2009). Systematic and integrative analysis of large gene lists using DAVID bioinformatics resources.. Nat Protoc.

[15] Kumar R, Blakemore SJ, Ellis CE, Petricoin EF, Pratt D (2010). Causal reasoning identifies mechanisms of sensitivity for a novel AKT kinase inhibitor, GSK690693.. BMC Genomics.

[16] Schlage WK, Westra JW, Gebel S, Catlett NL, Mathis C (2011). A computable cellular stress network model for non-diseased pulmonary and cardiovascular tissue.. BMC Syst Biol.

[17] Westra JW, Schlage WK, Frushour BP, Gebel S, Catlett NL (2011). Construction of a computable cell proliferation network focused on non-diseased lung cells.. BMC Syst Biol.

[18] Smith JJ, Kenney RD, Gagne DJ, Frushour BP, Ladd W (2009). Small molecule activators of SIRT1 replicate signaling pathways triggered by calorie restriction in vivo.. BMC Syst Biol.

[19] Toedter G, Li K, Sague S, Ma K, Marano C (2012). Genes associated with intestinal permeability in ulcerative colitis: Changes in expression following infliximab therapy.. Inflamm Bowel Dis.

[20] Laifenfeld D, Gilchrist A, Drubin D, Jorge M, Eddy SF (2010). The role of hypoxia in 2-butoxyethanol-induced hemangiosarcoma.. Toxicol Sci.

[21] Gentleman RC, Carey VJ, Bates DM, Bolstad B, Dettling M (2004). Bioconductor: open software development for computational biology and bioinformatics.. Genome Biol.

[22] Irizarry RA, Hobbs B, Collin F, Beazer-Barclay YD, Antonellis KJ (2003). Exploration, normalization, and summaries of high density oligonucleotide array probe level data.. Biostatistics.

[23] Smyth GK (2004). Linear models and empirical bayes methods for assessing differential expression in microarray experiments.. Stat Appl Genet Mol Biol.

[24] Benjamini Y, Hochberg Y (2000). Controlling the false discovery rate: A practical and powerful approach to multiple testing.. J Royal Statistic Soc.

[25] Kast-Woelbern HR, Dana SL, Cesario RM, Sun L, de Grandpre LY (2004). Rosiglitazone induction of Insig-1 in white adipose tissue reveals a novel interplay of peroxisome proliferator-activated receptor gamma and sterol regulatory element-binding protein in the regulation of adipogenesis.. J Biol Chem.

[26] Blander G, Bhimavarapu A, Mammone T, Maes D, Elliston K (2009). SIRT1 promotes differentiation of normal human keratinocytes.. J Invest Dermatol.

[27] Duval C, Muller M, Kersten S (2007). PPARalpha and dyslipidemia.. Biochim Biophys Acta.

[28] Chang BH, Chan L (2007). Regulation of Triglyceride Metabolism. III. Emerging role of lipid droplet protein ADFP in health and disease.. Am J Physiol Gastrointest Liver Physiol.

[29] Kersten S, Mandard S, Tan NS, Escher P, Metzger D (2000). Characterization of the fasting-induced adipose factor FIAF, a novel peroxisome proliferator-activated receptor target gene.. J Biol Chem.

[30] Vu-Dac N, Gervois P, Jakel H, Nowak M, Bauge E (2003). Apolipoprotein A5, a crucial determinant of plasma triglyceride levels, is highly responsive to peroxisome proliferator-activated receptor alpha activators.. J Biol Chem.

[31] Bell M, Wang H, Chen H, McLenithan JC, Gong DW (2008). Consequences of lipid droplet coat protein downregulation in liver cells: abnormal lipid droplet metabolism and induction of insulin resistance.. Diabetes.

[32] Grosskopf I, Baroukh N, Lee SJ, Kamari Y, Harats D (2005). Apolipoprotein A-V deficiency results in marked hypertriglyceridemia attributable to decreased lipolysis of triglyceride-rich lipoproteins and removal of their remnants.. Arterioscler Thromb Vasc Biol.

[33] Huang XS, Zhao SP, Bai L, Hu M, Zhao W (2009). Atorvastatin and fenofibrate increase apolipoprotein AV and decrease triglycerides by up-regulating peroxisome proliferator-activated receptor-alpha.. Br J Pharmacol.

[34] Lichtenstein L, Berbee JF, van Dijk SJ, van Dijk KW, Bensadoun A (2007). Angptl4 upregulates cholesterol synthesis in liver via inhibition of LPL- and HL-dependent hepatic cholesterol uptake.. Arterioscler Thromb Vasc Biol.

[35] Yoshida K, Shimizugawa T, Ono M, Furukawa H (2002). Angiopoietin-like protein 4 is a potent hyperlipidemia-inducing factor in mice and inhibitor of lipoprotein lipase.. J Lipid Res.

[36] Tall AR, Yvan-Charvet L, Terasaka N, Pagler T, Wang N (2008). HDL, ABC transporters, and cholesterol efflux: implications for the treatment of atherosclerosis.. Cell Metab.

[37] Brown MS, Goldstein JL (1997). The SREBP pathway: regulation of cholesterol metabolism by proteolysis of a membrane-bound transcription factor.. Cell.

[38] Aleksunes LM, Manautou JE (2007). Emerging role of Nrf2 in protecting against hepatic and gastrointestinal disease.. Toxicol Pathol.

[39] Gupta AK, Li B, Cerniglia GJ, Ahmed MS, Hahn SM (2007). The HIV protease inhibitor nelfinavir downregulates Akt phosphorylation by inhibiting proteasomal activity and inducing the unfolded protein response.. Neoplasia.

[40] Oyaizu T, Shikata N, Senzaki H, Matsuzawa A, Tsubura A (1997). Studies on the mechanism of dimethylnitrosamine-induced acute liver injury in mice.. Exp Toxicol Pathol.

[41] Gordon S, Akopyan G, Garban H, Bonavida B (2006). Transcription factor YY1: structure, function, and therapeutic implications in cancer biology.. Oncogene.

[42] Katso R, Okkenhaug K, Ahmadi K, White S, Timms J (2001). Cellular function of phosphoinositide 3-kinases: implications for development, homeostasis, and cancer.. Annu Rev Cell Dev Biol.

[43] Chalhoub N, Baker SJ (2009). PTEN and the PI3-kinase pathway in cancer.. Annu Rev Pathol.

[44] Gartel AL, Radhakrishnan SK (2005). Lost in transcription: p21 repression, mechanisms, and consequences.. Cancer Res.

[45] Rogue A, Lambert C, Josse R, Antherieu S, Spire C (2011). Comparative gene expression profiles induced by PPARgamma and PPARalpha/gamma agonists in human hepatocytes.. PLoS One.

[46] Romeo S, Yin W, Kozlitina J, Pennacchio LA, Boerwinkle E (2009). Rare loss-of-function mutations in ANGPTL family members contribute to plasma triglyceride levels in humans.. J Clin Invest.

[47] Goke B, Gause-Nilsson I, Persson A (2007). The effects of tesaglitazar as add-on treatment to metformin in patients with poorly controlled type 2 diabetes.. Diab Vasc Dis Res.

[48] Wilding JP, Gause-Nilsson I, Persson A (2007). Tesaglitazar, as add-on therapy to sulphonylurea, dose-dependently improves glucose and lipid abnormalities in patients with type 2 diabetes.. Diab Vasc Dis Res.

[49] Abourbih S, Filion KB, Joseph L, Schiffrin EL, Rinfret S (2009). Effect of fibrates on lipid profiles and cardiovascular outcomes: a systematic review.. Am J Med.

[50] Chiquette E, Ramirez G, Defronzo R (2004). A meta-analysis comparing the effect of thiazolidinediones on cardiovascular risk factors.. Arch Intern Med.

[51] Grundy SM, Vega GL (1987). Fibric acids: effects on lipids and lipoprotein metabolism.. Am J Med.

[52] Winkler K, Konrad T, Fullert S, Friedrich I, Destani R (2003). Pioglitazone reduces atherogenic dense LDL particles in nondiabetic patients with arterial hypertension: a double-blind, placebo-controlled study.. Diabetes Care.

[53] Sahin F, Sladek TL (2010). E2F-1 has dual roles depending on the cell cycle.. Int J Biol Sci.

[54] Ericsson H, Hamren B, Bergstrand S, Elebring M, Fryklund L (2004). Pharmacokinetics and metabolism of tesaglitazar, a novel dual-acting peroxisome proliferator-activated receptor alpha/gamma agonist, after a single oral and intravenous dose in humans.. Drug Metab Dispos.

